# Supplementary figures and images for: Sex-Differences in Traumatic Brain Injury in the Absence of Tau in Drosophila
Source: Genes (Basel). 2021 Jun 14;12(6):917. doi: 10.3390/genes12060917 (PMC8232113; doi:10.3390/genes12060917)

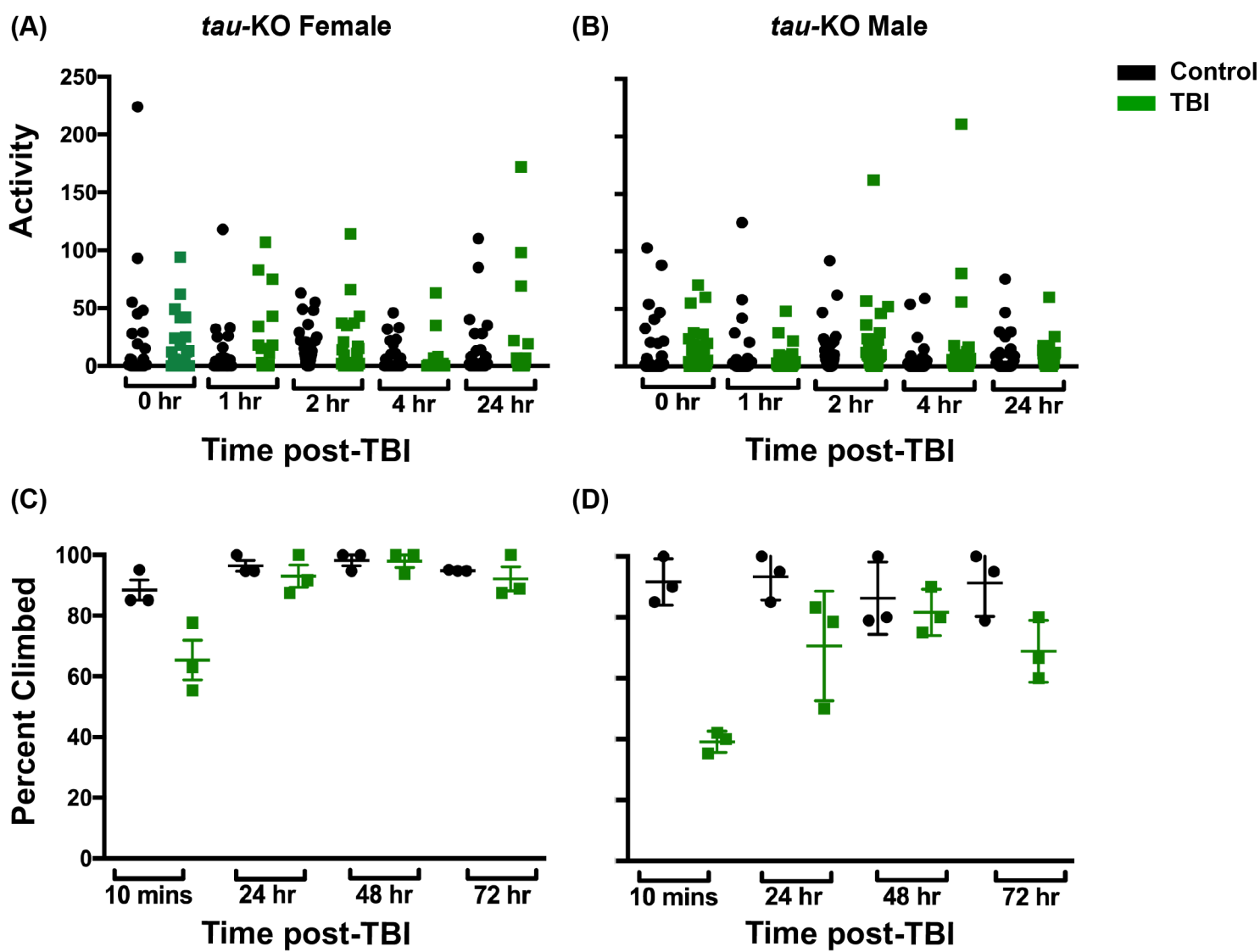

(A) Microscopic analysis of Tau-EGFP

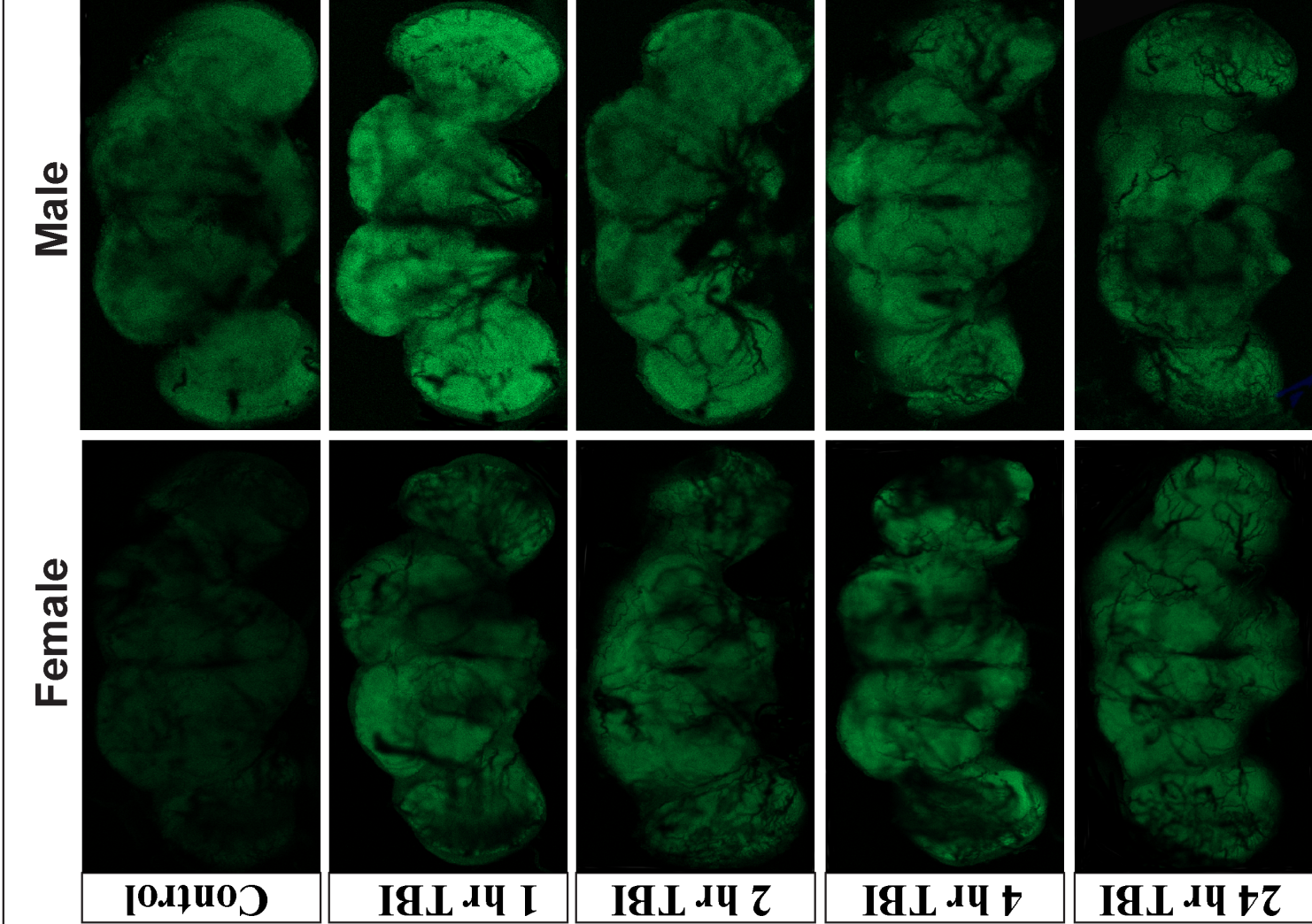

(B) Quantification of fluorescence intensity

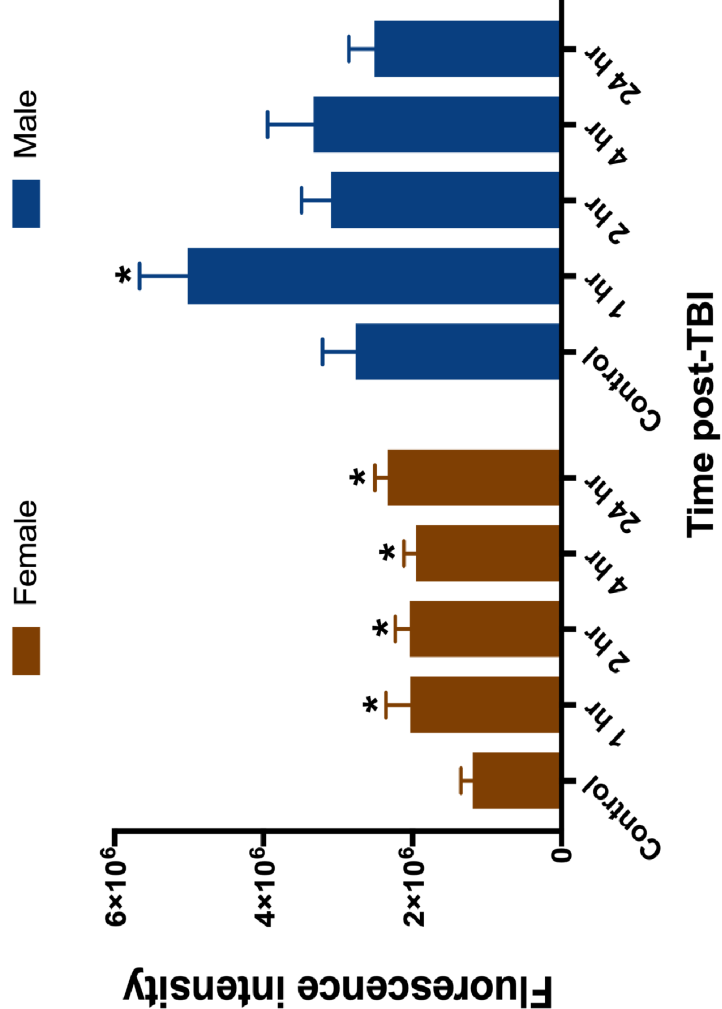

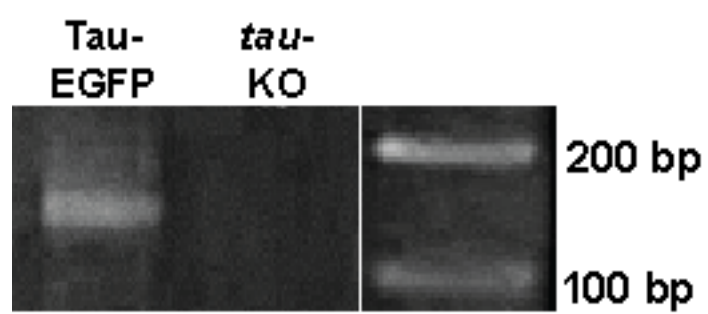

Supplement: Supplementary file 1 [file genes-12-00917-s001.zip › genes-1218083-supplementary.pdf]
